# Editorial: The Effects of Climate Change and Environmental Factors on Exercising Children and Youth

**DOI:** 10.3389/fspor.2021.690171

**Published:** 2021-05-28

**Authors:** Shawnda A. Morrison, Julien D. Périard, Patrick De Boever, Hein A. M. Daanen

**Affiliations:** ^1^Center for Climate Change and Active Children, Faculty of Sport, University of Ljubljana, Ljubljana, Slovenia; ^2^Faculty of Health, Research Institute for Sport and Exercise, University of Canberra, Canberra, ACT, Australia; ^3^Department of Biology, University of Antwerp, Antwerp, Belgium; ^4^Centre for Environmental Sciences, Hasselt University, Diepenbeek, Belgium; ^5^Department of Human Movement Sciences, Faculty of Behavioural and Movement Sciences, Amsterdam Movement Sciences, Vrije Universiteit Amsterdam, Amsterdam, Netherlands

**Keywords:** global warming, environmental epidemiology, hyperthermia, active play, COVID-19, hypoxia, pediatric health

The effects of climate change will exert both indirect (e.g., ecosystem disruption, air pollution, and changing disease-vector patterns) and direct (e.g., droughts, floods, wildfires, temperature increases) impacts on human health (Figure 1), especially in vulnerable populations like children (Helldén et al., 2021). How these factors affect physical activity (PA) in children is less frequently investigated. Indeed, child health is not prioritized in policy-making to the level required to reduce harm (Pegram and Colon, 2019). A recent scoping review concluded that children will experience high morbidity and mortality burden in the coming years because of climate change (Helldén et al., 2021).

**Figure 1:**
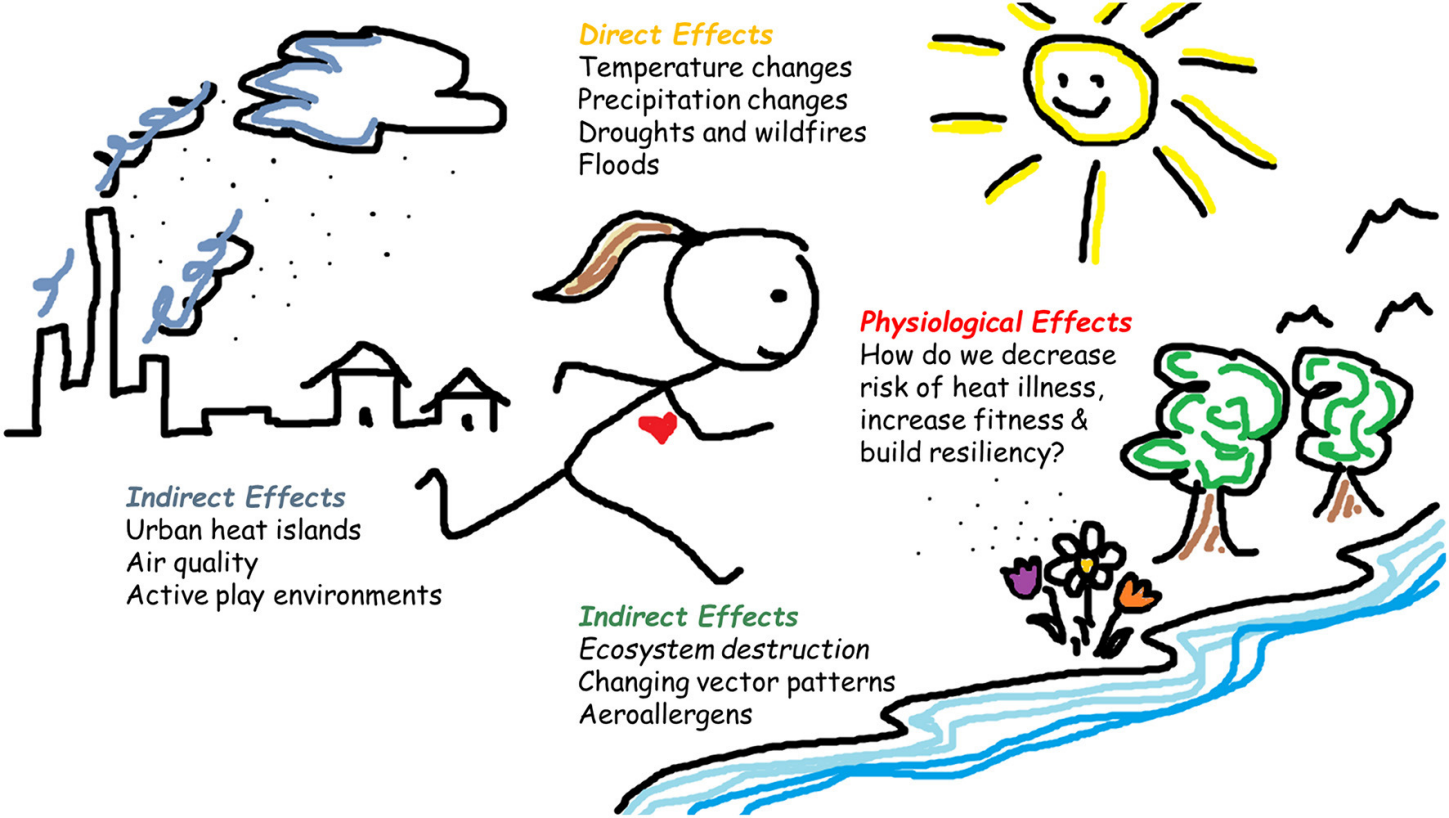
Effects of climate change on the health of exercising children. Climate change will require human adaptation to its direct (e.g., temperature changes, floods) and indirect interconnected effects (e.g., air pollution, changing disease vector patterns), especially in children, who are considered a vulnerable population and have not been the focus of recent scientific research or policy making to this point, despite clear evidence that children require special focus to reduce health risk. The heading “Physiological effects” refers to both positive and negative adaptations to climate change, from exposure to environmental factors like air pollution and heat, to being aware of the ongoing changes in body constitution of children (e.g., increasingly overweight) as a result of increased sedentary behavior and physical inactivity, which may directly affect heat tolerance and resiliency.

This Research Topic's goal was to collate investigations on how acute and chronic exposure(s) to environmental factors affect exercising children. Four papers were accepted on varied topics, including the influence of hypobaria on heart rate variability (Aebi et al.), outdoor PA trends in urban environments (Bao et al.), the effect of summer holidays on PA (Volmut et al.), and how to respond to pandemic self-isolation measures (Jurak et al.). In this editorial, three indirect effects of climate change and child health are explored, including (1) air quality and pressure, (2) urban design and active play, and (3) changing disease-vector patterns, and one direct effect, (4) high ambient temperatures.

## Air Quality and Pressure

Burning fossil fuels cause complex atmospheric emissions and negatively affects air quality. Compared to adults, children have rapidly-developing respiratory and immunological systems and smaller peripheral airways, making them particularly vulnerable to air pollution. Early-life exposure to air pollutants increases the risk of childhood health problems and predisposes them to chronic disease in adulthood (Perera, 2017). Young children, and especially the poor, suffer from environmental injustice because of these effects (Mathiarasan and Hüls, 2021). Ventilation rates increase during exercise. When this occurs, the inhaled air pollution dose increases, penetrating deeper into lung tissues, yet studies investigating PA and air pollution interactions have focused mainly on adults (Tainio et al., 2021). The composition and pressure of air also affect gas exchange in humans; environmental hypoxia occurs when there is a decrease in inspired oxygen pressure, which affects cardiac autonomic function. In their work, Aebi et al. tested young flight pilots. The authors confirmed that hypobaric hypoxia increases minute ventilation, decreases oxygen saturation, and affects heart rate variability parameters more so than under normobaric hypoxic conditions.

## Urban Design and Active Play

Modern society, especially in higher socio-economic status countries, often struggles to balance the idea of keeping children healthy and active vs. protecting them from serious harm. Many adults consider cities unsafe for children to play unsupervised, especially regarding outdoor PA and active play. In this way, adults may hinder children's natural ability to develop and learn, especially in outdoor environments. In their paper (Bao et al.) found that when children's PA spaces were fenced in, or featured excessive artificial design, uniform equipment, or the area lacked consideration of children's needs, outdoor PA was lower than in areas incorporating natural elements like green space, water, and sandy features. Measuring spontaneous, active play in children is notoriously difficult; however, objective devices like accelerometers can help quantify PA. Interestingly (Volmut et al.) reported that kids' overall PA decreased by ~18%, and physical *in*activity increased by 5.5% during summer holidays. Negative PA trends can be exacerbated as summers continue to warm, includng more extensive heatwaves. We should consider this evolution when kids have less access to structured, quality physical education minutes as they typically do during school time.

## Changing Disease Vector Patterns

Climate change will significantly affect disease-carrying vector distribution such that weather patterns will affect transmission and survival of infectious pathogens (Ahdoot and Pacheco, 2015). Therefore, the extraordinary impact the COVID19 pandemic has had on human movement restrictions will probably not be a one-off situation. From the early days of the pandemic, researchers sounded the alarm on the negative impact of isolation, confinement, and physical (in)activity on all persons (Burtscher et al., 2020) and children in particular (Morrison et al., 2020). In their article (Jurak et al.) outline the grave costs these restrictive measures have had on children's physical fitness, noting that their research group has observed the most significant decrease in child fitness in the >30 year history since systematic fitness testing began in Slovenia. The researchers detail a novel SLOfit Barometer system as a tool to engage in public health surveillance for the public and policymakers, hoping that other countries will use this model to create their systems of advocating for child health.

## High Ambient Temperatures

Children play, and when they do so in the heat, they may be at health risk as body temperatures rise (McGarr et al., 2021). Conversely, exercise in the heat leads to physiological adaptation, reducing heat strain. Unfortunately, information on the risks:benefits of active heat strain in children is lacking. During hot summer periods, which will occur more frequently because of climate change, children may be challenged to maintain stable body core temperatures due to slight differences in heat loss mechanisms compared with adults (Smith, 2019; Notley et al., 2020). Behavioral thermoregulation is compromised in small children who depend on supervisory control. It was recently shown that caretakers in daycare centers cannot accurately assess the children's thermal status, unlike their own thermal state (Folkerts et al., 2020). This poses an additional risk of heat strain for small children. Over two decades ago, research on heat strain in children was more prevalent. For example, researchers observed that hydration status is considerably better-maintained when children drink flavored vs. unflavored water (Bar-Or and Wilk, 1996). This type of research, with practical impact, should be revived now that it appears children can be at greater thermal risk during everyday life. Climate change, bush fires threatening schoolchildren's health (Requia et al., 2021), and inactivity caused by COVID19 (Ghosh et al., 2020), each justify a need to increase research focused on exercising children, similar to the attention recently given to occupational heat strain (Morris et al., 2020).

## Author Contributions

All authors listed have made a substantial, direct and intellectual contribution to the work and approved it for final publication.

## Conflict of Interest

The authors declare that the research was conducted in the absence of any commercial or financial relationships that could be construed as a potential conflict of interest.
